# Supplementary material for: Cell Hashing with barcoded antibodies enables multiplexing and doublet detection for single cell genomics
Source: Genome Biol. 2018 Dec 19;19:224. doi: 10.1186/s13059-018-1603-1 (PMC6300015; doi:10.1186/s13059-018-1603-1)
Supplement: Supplementary file 1 — Distribution of HTO UMIs per cell barcode. (PDF 6203 kb) [file 13059_2018_1603_MOESM1_ESM.pdf]

Figure S1

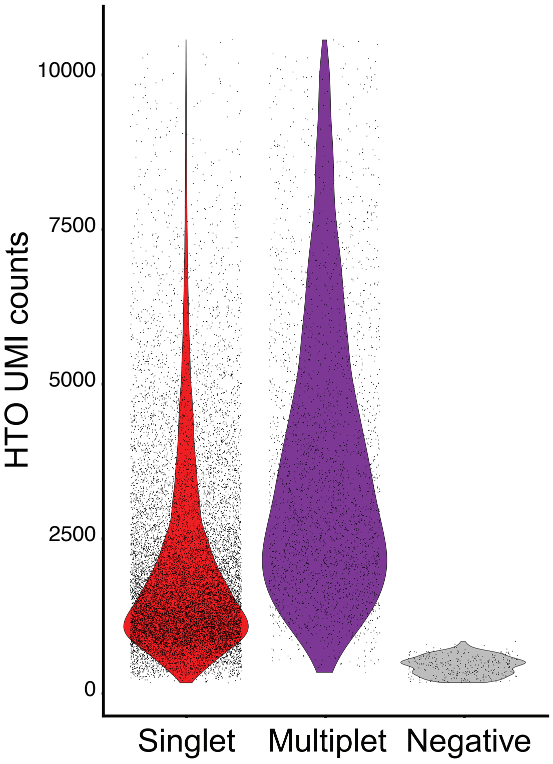

Figure S1 | Distribution of HTO UMIs per cell barcode. Distribution of HTO UMIs per cell barcode in cells that were characterized as singlets (red), multiplets (violet), or negatives (grey).

Figure S2

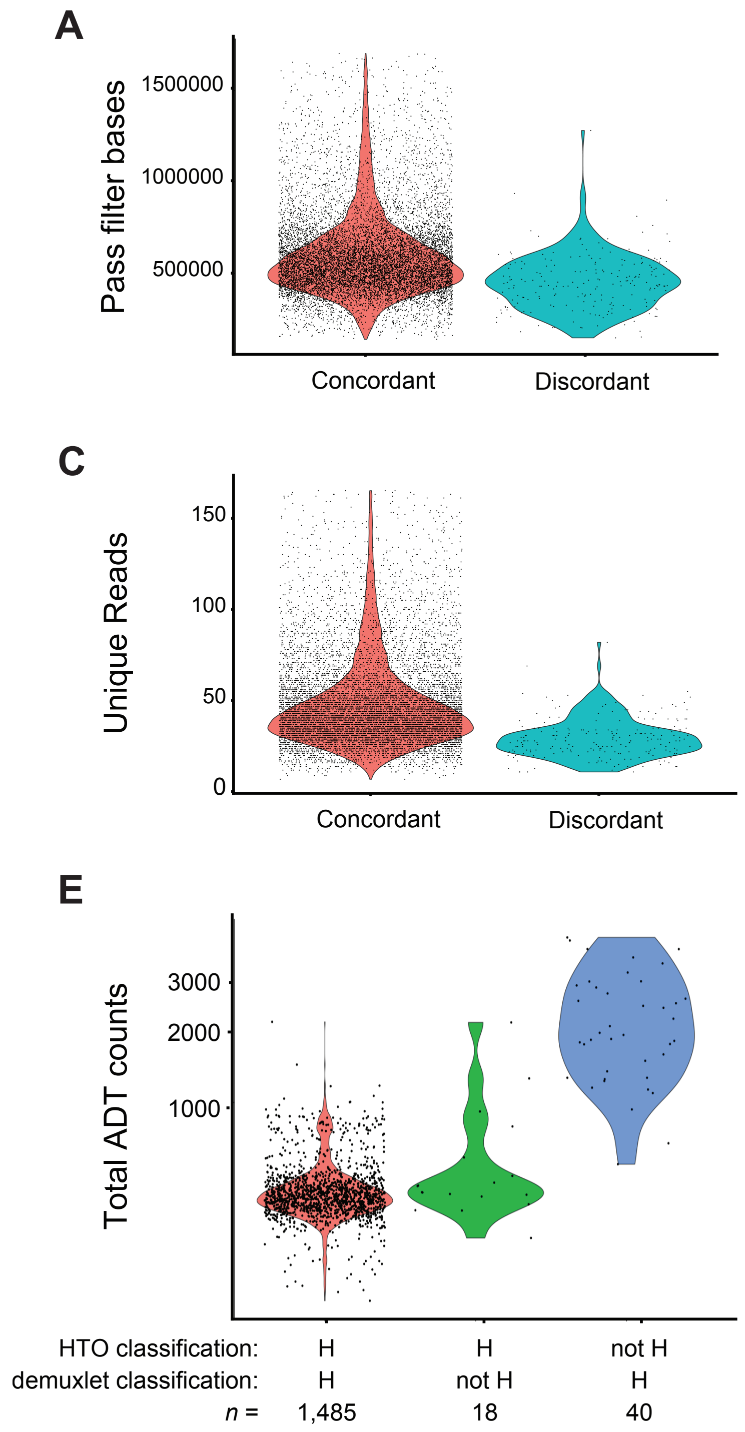

Figure S2 | Comparison of per-cell technical metrics between concordant and discordant classifications from Cell Hashing and demuxlet. (A-D) Represent outputs taken from demuxlet, and demonstrate that discordant calls had fewer reads, and fewer SNPs that could be used for classification. E) Antibody-derived tag (ADT) counts in concordant and discordant singlet classifications for donor H. Donor H was not stained with CITE-seq antibodies (Methods), and therefore, cells that originate from donor H should not exhibit robust ADT counts.

Figure S3

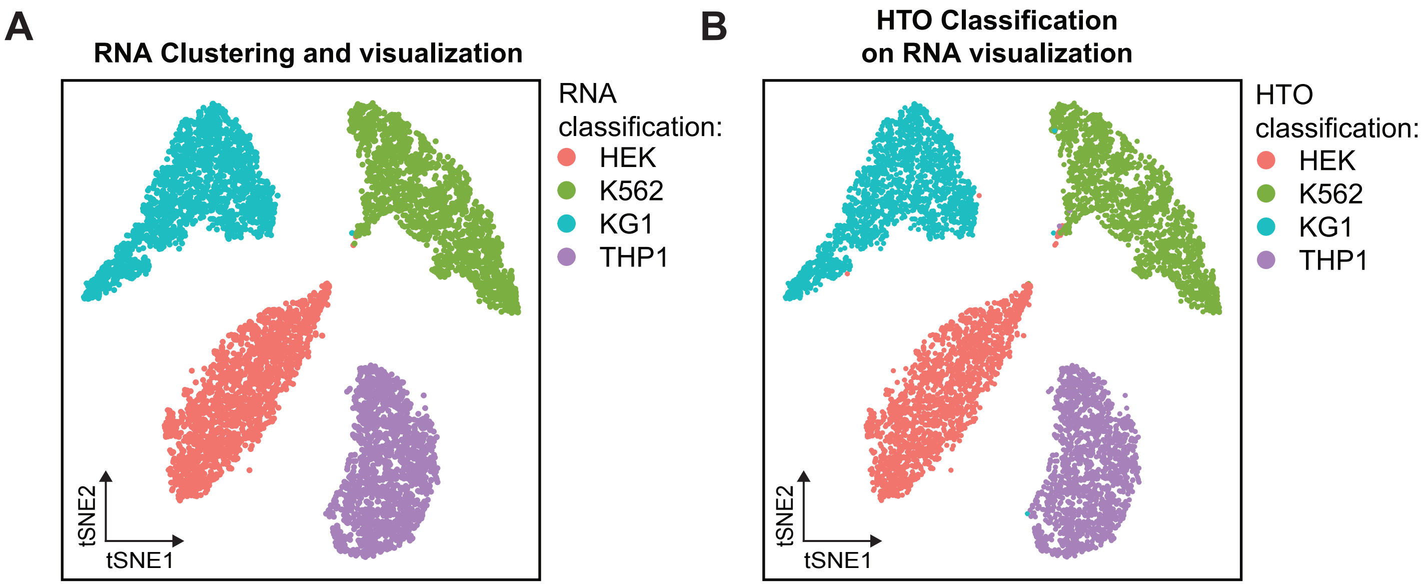

Figure S3 | Cell Hashing experiment with four transcriptomically distinct cell lines A) Transcriptome-based clustering of single-cell expression profiles reveals four distinct clusters, representing the HEK, THP1, K562 and KG1 cell lines in the experiment. B) Cells were visualized on the same two-dimensional tSNE plot computed from transcriptomes, but labeled based on their classification after HTO demultiplexing (each cell line was labeled with three distinct HTOs).

Figure S4

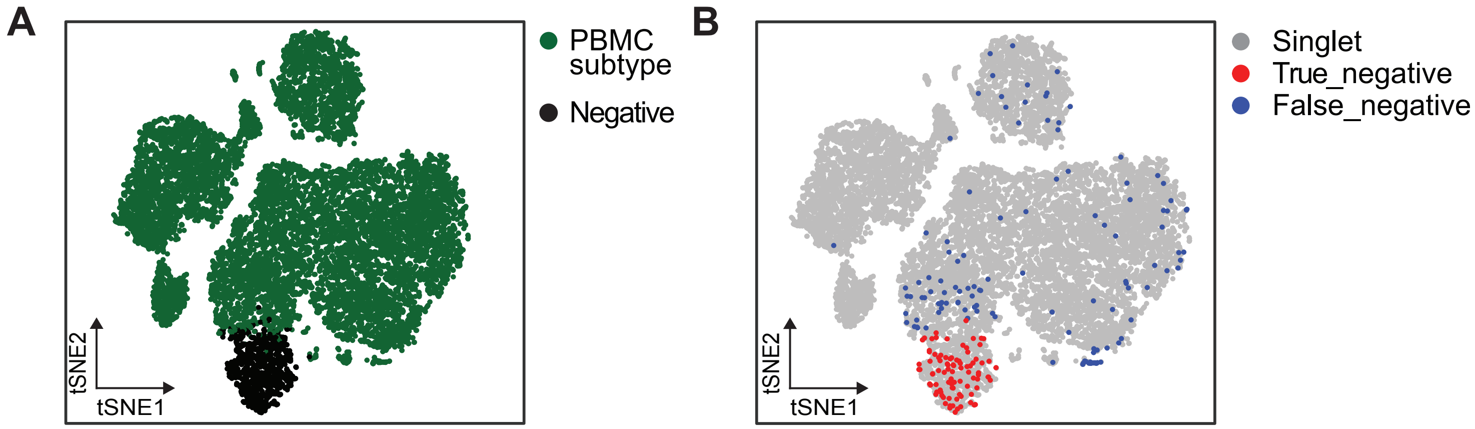

Figure S4 | Identification of 'false negative' barcodes A-B) Transcriptome-based clustering and visualization of all cell barcodes with >200 UMIs detected, that were classified as singlets or negatives based on HTO levels. We observed a transcriptomic cluster encompassing 'negative' barcodes that clustered separately from PBMC subtypes (A). (B) Cells are colored by their HTO classifications, revealing a small percentage of barcodes that were labeled as 'negative' but transcriptomically cluster with PBMC subtypes ('false negative' rate of 0.9%).

Figure S5

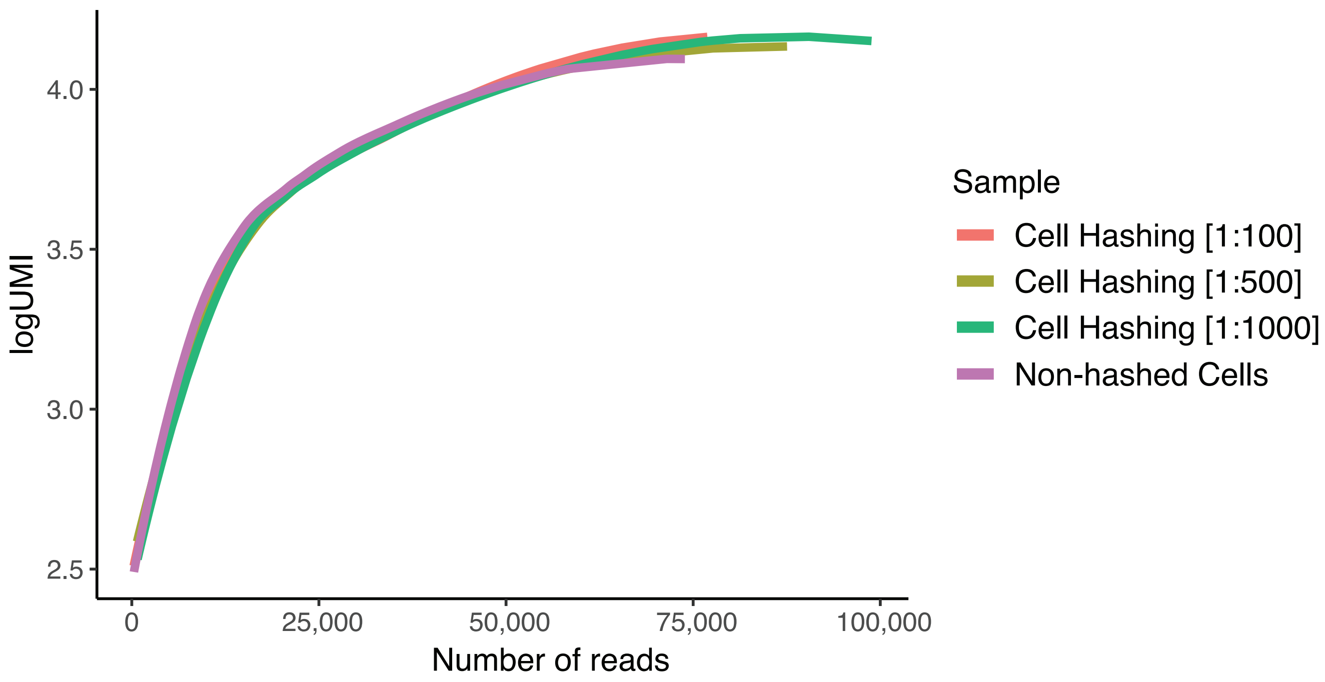

Figure S5 | Cell Hashing does not interfere with transcript capture. 'Saturation' curves showing the UMI observed per cell as a function of sequencing depth, grouped by concentrations of Cell Hashing antibodies used in a dilution series, compared to non-hashed control. We observe identical relationships across the dilution series, including in a control experiment performed without Cell Hashing.
